# An Overview of the Bioactivity of Spontaneous Medicinal Plants Suitable for the Improvement of Lung Cancer Therapies

**DOI:** 10.3390/pharmaceutics17030336

**Published:** 2025-03-05

**Authors:** Lidia-Ioana Virchea, Adina Frum, Cecilia Georgescu, Bence Pecsenye, Endre Máthé, Monica Mironescu, Mihai-Tudor Crăciunaș, Maria Totan, Ciprian Tănăsescu, Felicia-Gabriela Gligor

**Affiliations:** 1 Faculty of Medicine, “Lucian Blaga” University of Sibiu, Lucian Blaga Str. 2A, 550169 Sibiu, Romania; lidia.virchea@ulbsibiu.ro (L.-I.V.); maria.totan@ulbsibiu.ro (M.T.); ciprian.tanasescu@ulbsibiu.ro (C.T.); felicia.gligor@ulbsibiu.ro (F.-G.G.); 2 Faculty of Agriculture Sciences, Food Industry and Environmental Protection, “Lucian Blaga” University of Sibiu, Dr. Ion Rațiu Str. 7-9, 550012 Sibiu, Romania; cecilia.georgescu@ulbsibiu.ro (C.G.); monica.mironescu@ulbsibiu.ro (M.M.); 3 Institute of Nutrition Science, Faculty of Agricultural and Food Sciences and Environmental Management, University of Debrecen, Böszörményi Str. 128, 4032 Debrecen, Hungary; pecsenye.bence@agr.unideb.hu (B.P.); endre.mathe@agr.unideb.hu (E.M.); 4 Department of Life Sciences, Faculty of Medicine, Vasile Goldis, Western University from Arad, L. Rebreanu Str. 86, 310414 Arad, Romania; 5 Faculty of Sciences, “Lucian Blaga” University of Sibiu, Dr. Ion Rațiu Str. 5-7, 550012 Sibiu, Romania; mihai.craciunas@ulbsibiu.ro

**Keywords:** volatile oils, plant extracts, bioactive compounds, cancer, biological activity, cytotoxicity

## Abstract

Lung cancer is the second cause of death in the world, being the most common type of cancer. Conventional therapies are not always recommended due to the particularities of patients. Thus, there is a need to develop new anticancer therapeutic agents. Medicinal plants constitute a source of bioactive compounds with therapeutic potential in lung cancer. The purpose of our narrative review is to evaluate and summarize the main studies on the cytotoxic effects of ten medicinal plants and their extracts, volatile oils, and bioactive compounds. We have also included studies that reported protective effects of these natural products against chemotherapy-induced toxicity. Studies were identified by assessing five databases using specific keywords. The investigated natural products possess cytotoxic effects on lung cancer cell cultures. Several mechanisms of action have been proposed including cell death by apoptosis, necrosis or autophagy, cell cycle arrest, the modulation of signaling pathways (PI3K/Akt and MAPK), the inhibition of migration, invasion and metastasis, antiangiogenesis, and targeting inflammation. Different bioactive compounds exhibit protective effects against chemotherapy-induced toxicity. Studies have shown promising results. To develop new therapeutic agents useful in treating lung cancer, the plants included in this review should be more deeply investigated to reveal their molecular mechanisms of action.

## 1. Introduction

Nowadays, cancer has a major negative impact on society, public health, and the economies of many countries. One of six deaths is caused by cancer worldwide. In 2022, about 20 million new cancer cases were diagnosed and 9.7 million deaths were due to cancer. It is estimated that more than 35 million new cases will be diagnosed in 2050. Lung cancer is the most common type of cancer worldwide. In 2022, it had the highest incidence (2.5 million new cases) and mortality (over 1.8 million cancer deaths). This cancer is the most common cancer type among men and the second among women. Smoking is responsible for ¼ of the lung cancer cases. Other risk factors include air pollution, biomass fuels, and occupation [1].

The survival rate of patients with lung cancer (LC) is often decreased compared to those for other types of cancer because lung cancer is generally diagnosed in advanced stages and by that time, many patients already present distant metastasis [2]. There are two types of lung cancer: small cell lung cancer (SCLC) and non-small-cell lung cancer (NSCLC). NSCLC is more frequent (approximately 85% of the LC cases) compared to SCLC (10–15% of the LC cases) [3]. The correct diagnosis is crucial because these types of cancers would ultimately require individualized treatment [4]. A recent study reported a way to diagnose LC, but also to distinguish between NSCLC and SCLC, based on the analysis of the microbiota of bronchoalveolar lavage fluid [5]. The treatment of early stages of LC is surgery. After surgery, adjuvant therapy consisting of chemo-/radio-/immuno-/targeted therapy is recommended [3]. Chemotherapy is also used in advanced stages of LC. Some chemotherapeutic drugs exert beneficial effects in NSCLC, like cisplatin, carboplatin, paclitaxel, docetaxel, gemcitabine, vinorelbine, etoposide, and pemetrexed, and in SCLC, such as cisplatin/carboplatin and etoposide/irinotecan or topotecan and lurbinectedin [3]. Immunotherapy and targeted therapy reduced mortality among advanced-stage NSCLC patients [6]. However, many shortcomings in treating LC must be addressed to increase the efficacy of the applied treatment [7]. Moreover, chemotherapy is limited due to its toxicity, side effects [8], low bioavailability, low therapeutic indices, high doses needed [9], or resistance to treatment [10]. Immunotherapy, despite its effectiveness in treating cancers, presents multiple adverse effects (autoimmune diseases, gastrointestinal reactions, hepatotoxicity, and skin reactions). In addition, immunotherapy is very expensive and its specificity is questionable [11]. In some cases, conventional therapy is not indicated due to the patients’ specific features, such as comorbidities, age, or adverse events of the therapy. In these cases, an additional option could be the consumption of herbal medicines [2]. However, we do emphasize that natural products cannot replace conventional therapy. The therapeutic effects of medicinal plants are intensively studied and as the emerging system biology type of knowledge becomes a great asset for health care providers, they would feature an increasing interest in the application of medicinal plants as adjuvant treatment.

For many years, researchers have been focused on developing new anticancer therapies based on plant sources [12,13]. Medicinal plants have provided several anticancer compounds used in therapy, such as vincristine, paclitaxel, and docetaxel [13]. Traditional and alternative medicine is also used in cancer treatment and several studies have shown that medicinal plants are efficient in the treatment of LC [14]. A recent study highlighted the efficacy of cycloastragenol on NSCLC cell lines [15]. Plant extracts and their bioactive compounds have cytotoxic activity by increasing apoptosis [15,16,17,18,19,20], interfering with cell cycle regulation [19] and the inhibition of angiogenesis [14]. Medicinal plant extracts slowed down the development of metastases of different cancers (breast, liver, colon, gastric) by inhibiting matrix metalloproteinases (MMPs) [14]. In addition, the side effects and costs of plant-based anticancer compounds appear to be more reasonable compared to conventional therapy [20].

The cytotoxic and protective effects of natural products are most likely due to several phytonutrients, which, alone or in combination, may exert different physiological effects. In addition, some compounds can initiate both cytotoxic and/or cytoprotective actions. Phenolic compounds are well known for their antioxidant activity. They can protect the cells against damage induced by oxidative stress generated by chemotherapy, and also, they can induce cytotoxicity [21].

**Figure 1:**
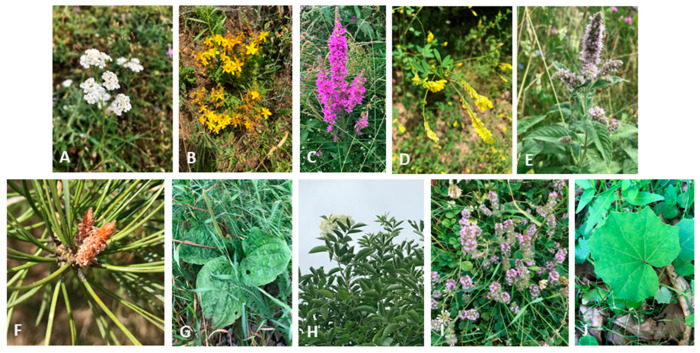
Spontaneous medicinal plants with beneficial effects on lung cancer ((**A**) *Achillea millefolium* L., (**B**) *Hypericum perforatum* L., (**C**) *Lythrum salicaria* L., (**D**) *Melilotus officinalis* L., (**E**) *Mentha longifolia* L., (**F**) *Pinus sylvestris* L., (**G**) *Plantago major* L., (**H**) *Sambucus nigra* L., (**I**) *Thymus serpyllum* L., and (**J**) *Tussilago farfara* L.).

The aim of this narrative review is to assess the in vitro and in vivo studies related to the cytotoxic effects of medicinal plants from the spontaneous mountain flora on LC and the protective effect of these natural products against the toxicity induced by chemotherapy and to provide an overview of this topic. Ten plants are considered, namely *Achillea millefolium* L. (Figure 1A), *Hypericum perforatum* L. (Figure 1B), *Lythrum salicaria* L. (Figure 1C), *Melilotus officinalis* L. (Figure 1D), *Mentha longifolia* L. (Figure 1E), *Pinus sylvestris* L. (Figure 1F), *Plantago major* L. (Figure 1G), *Sambucus nigra* L. (Figure 1H), *Thymus serpyllum* L. (Figure 1I), and *Tussilago farfara* L. (Figure 1J). This review is a starting point for experimental studies conducted on these plant species. The selection of plants included in this review was based on scientific evidence regarding their anticancer effects [20,22,23,24,25] and protective properties against the chemotherapy-induced toxicity [26,27,28] of extracts, volatile oils, and bioactive compounds, with some studies reporting the mechanisms of action that produce these effects [19,29,30,31]. In addition, the plants included in the paper are well known in traditional medicine for treating a variety of conditions [32,33,34,35,36,37,38,39,40,41]. Another selection criterion was the abundance of these species in the spontaneous flora, which could be exploited for the development of new innovative pharmaceutical products. This review is relevant in the current context as it provides researchers with an overview of recent studies in the scientific literature. To the best of our knowledge, there have been no other reviews that have summarized information on the cytotoxic effects of all these plant species and their possible application as adjuvant treatment, and we consider that this review can be the basis of future research on the therapeutic effects of these plants.

## 2. Methodology

For this narrative review, in vitro and in vivo studies related to the cytotoxic effects on LC, and protective effects against toxicity induced by chemotherapy, of essential oils, extracts, and bioactive compounds from ten plants (*Achillea millefolium* L., *Hypericum perforatum* L., *Lythrum salicaria* L., *Melilotus officinalis* L., *Mentha longifolia* L., *Pinus sylvestris* L., *Plantago major* L., *Sambucus nigra* L., *Thymus serpyllum* L., and *Tussilago farfara* L.) were analyzed from five databases: Web of Science, Scopus, PubMed, ScienceDirect, and Google Scholar. Only studies published between 2000 and 2024 were included. Main keywords used in research were combinations of the scientific name of each investigated plant, the names of bioactive compounds, “lung cancer”, “cytotoxic effect”, the names of cell lines, and “protective effect”. Only articles written in English were included. The quality of the included studies was assessed by the availability of details about medicinal plants, extract type, cell lines, tested concentrations, and the reporting of results.

This review consists of seven sections. In the Section 1, Section 2 and Section 3 we presented an introduction about LC, its conventional therapy and the need to investigate new treatment options based on natural products, the methodology and the most important cell lines used in LC research. Section 4 presents information about the selected medicinal plants and summarizes the main findings of recent studies that investigated the cytotoxicity against LC and normal cell lines, but also the protective effect against chemotherapy-induced toxicity reported for each plant species. Section 5 describes the parameters involved in establishing cytotoxicity. Section 6 focuses on the most important mechanisms of action responsible for the cytotoxic effects of natural products, the study ending with the most relevant conclusions.

## 3. Lung Cancer Cell Lines Used in Cytotoxicity Studies

The development of lung cell line cultures has made a high contribution in LC research. Nowadays, a collection of over 200 LC cell lines is available, and these cells are worldwide used in in vitro studies. The relevance of using LC cell lines in LC research is due to the high similarity of these cells with the tumors from which they are derived. The accuracy of the studies can be diminished by misidentifying or contaminating cells with mycoplasma, viruses, or other cell cultures [42].The most lung cancer cell lines used in studies to assess the cytotoxicity of the ten plants included in this review can be classified by their types into NSCLC (such as A549 [43], NCI-H460 [44], NCI-H1975 [45], H1299 [46], RERF-LC-kj, QG-56 [47], H157 [48], CLI-5-F4 [17], LU-1 [49], and PC-9 [20]) and SCLC cell lines (such as QG-90 and PC-6 [47]). In the majority of the studies that reported the cytotoxic potential of the considered medicinal plants, the A549 LC cell line was used. A549 cells are squamous, basal human alveolar epithelial cells. These cells were reported by Giard et al. in 1972, and they were obtained from cancerous lung tissue taken from a Caucasian man. The A549 cells play a role in the diffusion of electrolytes and water through the pulmonary alveoli. In vivo, they grow in an adherent monolayer [50]. The A549 cells are used as part of a model for the study of LC [51]. This cell line is used in many studies that focus on testing the cytotoxic effects of drugs, bioactive plant compounds, plant extracts, or nanoparticles (NPs) with plant extracts, as well as in those elucidating the mechanism of action responsible for this effect [18,22,52].

## 4. Medicinal Plants with Cytotoxic and Protective Effects on Lung Cancer

### 4.1. Achillea millefolium *L.*

*Achillea millefolium* L. (yarrow) is a perennial herbaceous plant belonging to the Asteraceae family [53]. The Asteraceae family contains over 1600 genera and 25,000 species [54]. The *Achillea* genus includes over 130 species. It is indigenous to the Northern Hemisphere. It is found in Europe, Asia, and North America [53]. *Achillea millefolium* L. grows in home gardens, pathways, crop fields, or mountain meadows [44]. The plant can reach 50 cm in height. The bipinnate or tripinnate leaves are 5–20 cm in length; they are placed spherically from the bottom to the middle of the stem. The flowers are grouped in corymb-type inflorescences containing flattened ovoid heads and placed at the top of the stems and branches. The fruits are 2 mm in length, of a shiny achene type, and oblong, with broad winged margins and no pappus [55].

Wild *Achillea millefolium* L. contains a variety of phenolic compounds such as caffeoylquinic acid derivatives (3-O-, 4-O-, and 5-O-caffeoylquinic acids), dicaffeoylquinic acids (3,4-O-, 3,5-O-, and 4,5-O- dicaffeoylquinic acids), flavones (apigenin and luteolin derivatives), and flavonol derivatives (quercetin and kaempferol derivatives) [44]. *Achillea millefolium* L. is a source of volatile oil in which thymol, borneol, limonene, carvacrol, and α-pinene are the most abundant compounds [56]. Phenolic compounds (flavonoids and phenolcarbonic acids) are the most representative, and they are responsible for effects such as antiplasmodial [57], antioxidant [57,58], and antipyretic [59].

*Achillea millefolium* L. volatile oil has antioxidant, antimicrobial, and anti-inflammatory effects [56]. In traditional medicine, the plant is used to manage headache, hemorrhage, hemorrhoids, high blood pressure, respiratory issues (influenza, pneumonia, and cough), kidney disorders, gastrointestinal disorders, and skin ailments [32]. The cytotoxic effects of both pure *Achillea millefolium* L. extracts as well as the extracts incorporated in NPs on SCLC, NSCLC, and normal cell lines have been investigated [18,23,29,43,44,45,47,48,52,60,61,62]. Variations in the half-maximal inhibitory concentration (IC_50_) values have suggested that the cytotoxic effect depends on the origin of the vegetal product and the extraction method used. In some studies, the cytotoxicity was attributed to different bioactive components, especially phenolic compounds, which were the main constituents of the extracts [29,43,44,60]. Other authors analyzed the cytotoxic and antiproliferative effects of bioactive compounds of *Achillea millefolium* L. on LC cell lines. The effects of different extracts and bioactive compounds obtained from *Achillea millefolium* L. on LC and normal cell lines are summarized in Table 1. Also, some major bioactive compounds from extracts are listed.

A study in which 56 patients were involved reported that the use of a mixture of *Achillea millefolium* L. distillate (10 kg of flowers in 50 L of water) and mouthwash, administered four times a day for 14 days, had a superior effect on chemotherapy-induced oral mucositis compared to the routine administration of the mouthwash alone [63]. *Achillea millefolium* L. extracts have shown protection against the toxic effects experimentally induced by chemotherapeutic agents in rats (Table 2). The protective effect of ethanol extracts of *Achillea millefolium* L. against vascular toxicity can be attributed to polyphenols that possess antioxidant and anti-inflammatory properties [26].

In some research studies, medicinal plants are used for early diagnosis, staging, or treating cancer. Therefore, Yurt Kilcar et al. investigated the in vitro incorporation of ^125^I radiolabeled *Achillea millefolium* L. compounds into the A549 cell line. The results showed that the luteolin marked with ^125^I led to increasing the percentage of incorporation from 15.4% at 30 min to 21.4% at 120 min. ^125^I alone did not have any effect on the analyzed cells since the incorporation percentage was approximately 0.5% [12].

### 4.2. Hypericum perforatum *L.*

*Hypericum perforatum* L. (St. John’s wort) is a flowering plant species that belongs to the Hypericaceae family. This family contains about 55 genera and over 1000 species [67]. It is native to Europe and Asia [68]. It grows in meadows, fields, roadsides, waste areas, mines, and abandoned quarries. It is an herbaceous shrub, freely branched, 40–80 cm in height. The stem and the branches are covered by dense leaves. The leaves are elongated, with smooth edges, 1–3 cm in length and 0.3–1 cm in width. In the light, the leaves show translucent spots. The plant blooms with abundant yellow flowers, with dark spots on the edges of the petals. When picking up the flowers, a red pigment is released [69].

*Hypericum perforatum* L. contains fatty acids (oleic, palmitic, linoleic, and stearic) and tocopherols [70]. Methanol extracts of *Hypericum pretforatum* L. are rich in phenolic compounds such as phenolic acids (quinic acid derivatives, chlorogenic, and neochlorogenic acids and glucosides of caffeic and vanillic acids), flavanols (catechin and epicatechin), flavonols (hyperoside, quercitrin, quercetin, and rutin), naphthodianthrones (hypericin and pseudohypericin) and phloroglucinols (hyperforin and adhyperforin) [71]. Sesquiterpenes (β-caryophyllene, caryophyllene oxide, and β-selinene), alcohols (spathulenol, alpha cadinol, and phytol), and fatty acids (pentadecanoic and palmitic) are the major constituents of *Hypericum perforatum* L. volatile oil [72].

*Hypericum perforatum* L. has antimicrobial, anti-inflammatory, antitumor, and neuroprotective effects. It could be used in a potential treatment of neurodegenerative disorders such as Alzheimer’s and Parkinson’s diseases. The plant is known in traditional medicine to treat depression, pain, and hemorrhoids [33].

Studies reporting the cytotoxic effects of *Hypericum perforatum* L. extracts and bioactive compounds on LC and normal cell lines are listed in Table 3. Also, some major bioactive compounds from extracts are listed.

*Hypericum perforatum* L. extracts and hyperforin demonstrated anticancer properties. Hyperforin has anticancer activity due to its protonophore effect. In the case of normal cells, the difference in pH between the extracellular and intracellular spaces is small, while in the case of cancer cells, this difference is significant. The extracellular space is more acidic, and in the cytoplasm, the environment is more basic. Hyperforin causes H^+^ influx that results in a decrease in cytosolic pH and changes in biochemical processes in the cancer cell [68]. After treatment with hyperforin, a decrease in lung metastases was observed in mice in a study [80]. Flavonoids and tannins may be responsible for St. John’s wort’s anticancer activity [81].

Another study investigated the liver toxicity of the co-administration of lorlatinib (a medicine used to treat NSCLC) with *Hypericum perforatum* L. in Cynomolgus monkeys. *Hypericum perforatum* L. contains bioactive compounds known to moderately increase the activity of cytochrome (CYP) P450 isotype 3A in monkeys and humans, but also acts as a pregnane X receptor agonist in humans. The animals did not show clinical signs of liver toxicity. No changes were observed for the co-exposure to lorlatinib or its metabolite with *Hypericum perforatum* L. The systemic concentration of hyperforin was below the limit of quantification or was quantitated for a limited time, whether given alone or in combination with lorlatinib. The 4b-HC/C ratio increase was 1.1-fold, signifying weak CYP activation in monkeys. The reason for these observations may have been the too-low concentration of hypericin in the tested product (capsules with 0.3% hypericin) [82]. Eichkorn et al. presented a case study of an NSCLC patient who received radiotherapy for brain and bone metastases after a treatment with docetaxel and ramucirumab. During radiotherapy, the patient used a topical product with *Hypericum perforatum* L., which was administered on the scalp. They observed that, in combination with radiotherapy, the *Hypericum perforatum* L. product caused severe skin toxicity in the form of folliculitis [83]. Satia et al. reported that there was no association between LC risk and the administration of *Hypericum perforatum* L. supplements [81]. Furthermore, the *Hypericum perforatum* L. extract exhibited a protective effect against nephrotoxicity induced by cisplatin in rats [84]. It also protects against osteotoxicity induced by methotrexate in MLO-4Y cells [85]. *Hypericum perforatum* L. ameliorates irinotecan-induced toxicity in rats [86,87] and protects against bone marrow toxicity induced by irinotecan [88].

Usually, cancer patients do not seek the advice of their oncologists when deciding to take dietary or herbal supplements. As a result, there have been many reports of interferences between cancer drugs and natural remedies [89]. A dose of 900 mg/day of *Hypericum perforatum* L. administered orally for 18 days was observed to decrease the plasma level of the active metabolite of irinotecan (SN-38) by 42% [90]. Irinotecan is a prodrug metabolized in the liver by carboxylesterase to its active form (SN-38). Another route of metabolism of irinotecan occurs under the action of CYP3A4 and leads to inactive forms [91]. Therefore, the decrease in the plasma concentration of the active metabolite can be explained by the stimulation of CYP3A4 by the compounds in St. John’s wort extract, which favors the transformation of the drug into inactive forms [90]. A quantity of 300 mg of St. John’s wort extract (with a standardized concentration of hypericin and hyperforin) administered three times daily for 14 days resulted in decreased plasma concentration and toxicity and increased the elimination of docetaxel due to CYP3A4 activation [92]. *Hypericum perforatum* L. also decreases the plasma level of cyclophosphamide [93]. Most anticancer drugs are metabolized, being substrates for metabolizing enzymes and transporters. Interferences between anticancer drugs and herbal supplements are caused by enzyme activation or inhibition. When natural remedies are used concurrently with medication, the plasma concentration of the drug should be monitored and possible toxic effects must be assessed [89]. The stimulation of metabolism and increased elimination of active forms of chemotherapy drugs observed in the studies described above can be attributed to hyperforin. Hyperforin activates the pregnane X receptor, which forms a complex with the retinoid X receptor. Next, the complex binds to the DNA response element, influencing the transcription. Therefore, the production of CYP3A4 is stimulated, which leads to a decrease in the plasma level of the active form of the drug and to the failure of the therapy [94]. Importantly, the detection of these interferences should be considered a critical safety measure that cannot be ignored at any cost.

### 4.3. Lythrum salicaria *L.*

*Lythrum salicaria* L. (purple loosestrife) is a perennial herbaceous plant belonging to the Lythraceae family. The Lythraceae family consists of 32 genera and 620 species. *Lythrum salicaria* L. is native to Europe, Asia, northwest Africa, and southeastern Australia. The plant prefers moist places. It grows in banks and wet meadows. The plant can reach 30–150 cm in height; the stems are erect; the leaves are opposite or appear in threes in whorls, lanceolate; and the reddish-purple flowers are grouped in cymes forming long terminal spikes [95].

In *Lythrum salicaria* L. aerial-part extracts, coumarins, ellagic acid derivatives, triterpenes, steroids, 5-hydroxypyrrolidin-2-one, phytol, and dodecanoic acid have been identified [96]. Methanol extracts of *Lythrum salicaria* L. contain phenolic compounds such as vitexin, ellagic acid, gallic acid, orientin, and isoorientin [97].

In traditional medicine, some of the effects of this plant, such as antihemorrhagic effects, astringent effects, and effects against pathogenic bacilli, are well documented. The plant is used in the treatment of diarrhea, dysentery, gastrointestinal hemorrages, nose bleeding, and infected wounds. Scientific studies have reported antimicrobial, anti-inflammatory, antioxidant, antidiarrheal, anticoagulant, pro-coagulant, antinociceptive, hypo-glycemic [34], bronchodilatory, and antitussive effects [98].

Some bioactive compounds from *Lythrum salicaria* L. have been reported to possess cytotoxic effects on colon, breast, and leukemia cell lines [96]. There were no studies in the analyzed scientific literature that reported the cytotoxic effects of *Lythrum salicaria* extracts on LC cell lines, but some of the bioactive compounds also found in *Lythrum salicaria* L. exhibited anticancer properties in LC. Liu et al. reported that vitexin decreased the viability of the human NSCLC cell line A549 [99]. Ellagic acid inhibited LC tumor growth [100] and induced the apoptosis of the A549 NSCLC cell line [101]. Gallic acid also decreased the viability of the A549 cell line [102].

### 4.4. Melilotus officinalis *L.*

*Melilotus officinalis* L. (yellow sweet clover) is a biennial robust herbaceous plant species belonging to the Fabaceae family [103]. The Fabaceae family has approximately 770 genera and 19,500 species [104]. It is native to Europe and Asia, and it also grows in America [105]. It grows in meadows and crops [106]. Its height can reach more than 1 m; the leaves are trifoliate; its leaflets are oblong, obovate, or oblanceolate; the yellow flowers are grouped in loose racemes, and they form ovoid, transversely rough, brown pods when ripe. The seeds are oval, smooth, yellow–green in color, and 2–3 mm in diameter [103].

*Melilotus officinalis* L. contains daucosterol, androsin, p-hydroxycinnamic and p-hydroxybenzoic acids [107], p-hydroxybenzoic acid glycosides, organic acids (salicylic, fumaric, and caffeic acids), flavonoids (luteolin and quercetin), coumarin, betaine [108], and carotenoids (violaxanthin, lutein 5,6-epoxide, lutein, and β-carotene) [109]. The hexane extract of *Melilotus officinalis* seeds contains coumarins, fatty acids ((9Z,12Z)-octadecadienoic, 14-methylpentadecanoic, (9E)-octadecenoic, linoleic, octadecanoic, oleic, palmitic, linolenic, eicosanoic, and 18-methylnonadecanoic), and 5-dodecyldihydrofuran-2(3H)-one [110].

In traditional medicine, *Melilotus officinalis* L. is known for its therapeutic effects such as its anti-inflammatory, emollient, tonic, antiflatulent, and carminative effects. It is used against pain, diabetes, infections, and malaria. Biological activities of the yellow sweet clover such as its antimicrobial [35], antioxidant, anti-inflammatory, and antitumor [108] activities have been reported. Studies have reported antitumor effects attributed to different compounds identified in *Melilotus officinalis* L. [108,111], but no evidence related to LC has been found. However, there were studies that attested the anticancer effects on LC cell lines of the bioactive compounds identified even in *Melilotus officinalis* L., such as rutin [112], chlorogenic acid [113], kaempferol [114], or quercetin [115].

Faid investigated the protective effect of a *Melilotus officinalis* L. extract against the pulmonary and cardiac toxicity produced by cisplatin. This study proved that the administration of *Melilotus officinalis* L. extract to rats, in doses of 100 mg/kg body weight and 200 mg/kg body weight, before the treatment with cisplatin improved the results of biochemical and histopathological tests related to pulmonary and cardiac toxicities [116].

### 4.5. Mentha longifolia *L.*

*Mentha longifolia* L. (wild mint) is an aromatic perennial herb [117] belonging to the Lamiaceae family [118]. This family comprises over 230 genera and 7000 species [119]. It is found in the Mediterranean area, Europe, North Africa, and Australia [36]. It grows on the banks of rivers and lakes; in ditches, meadows, forest edges, and wastelands; and along roads [120]. The stems are erect, up to 150 cm high; the opposite leaves are soft and hairy, having a green to gray color, and the shapes of the leaves are ovate and lanceolate, with slightly toothed edges. At the top of the stems, there are small white or light purple flowers grouped in clusters [121].

*Mentha longifolia* L. aerial-part extracts contain numerous secondary metabolites, such as phenolic compounds (catechin; cinnamic, benzoic, hydroxybenzoic, coumaric, chlorogenic, and gallic acids) and volatile constituents (1-isopropyl-4-methyl-1,4-cyclohexadiene; pulegone; phenol, 2-methyl-5-(1-methylethyl); and caryophyllene) [117]. The volatile oil is rich in cis-/trans-menthone, pulegone, 1,8 cineole, and menthol. Those compounds are responsible for antioxidant and antimicrobial effects. It also has anti-inflammatory and very low anticancer effects [118]. It can be used in the management of diseases related to the respiratory system (asthma, dyspnea, and common cold) and it can alleviate symptoms such as cough [36,122].

Studies showing the cytotoxicity of *Mentha longifolia* L. extracts and volatile oils against LC and normal cell lines are summarized in Table 4. Also, some major bioactive compounds are listed.

An ethanol extract of *Mentha longifolia* L. (250 and 500 mg/kg, 10 days) exhibited cardio-protective effects against the cardiac toxicity induced by cyclophosphamide in rats. The protective effect can be attributed to flavonoids, tannins, and saponins [125].

### 4.6. Pinus sylvestris *L.*

*Pinus sylvestris* L. (Scots pine) belongs to the Pinaceae family. This family consists of 11 genera and about 200 species [126]. *Pinus sylvestris* L. is native to Eurasia, but it grows especially in Northern and Eastern Europe [127]. It grows in bogs, rocky areas, and mixed forests [128]. Its height can reach up to 45 m; the leaves are green to greenish-blue, acicular, with a characteristic odor, 3–5 cm in length; they are arranged alternately or spirally [127]; and the bark is reddish brown [37].

The leaves contain volatile oils rich in α-/β-pinene, limonene, bornyl acetate, α-/β-felandrene, δ-3-carene, resin, and bitter principles [37,129,130]. Phenolic compounds, such as phenolic acids (cinnamic, syringic, chlorogenic, and ferulic acids), flavonoids ((+)-catechin, rutin, and quercetin), and stilbenes (resveratrol), have also been identified in *Pinus sylvestris* L. extracts [131]. Pine volatile oil has numerous applications in medicine due to its antiviral, antiallergic, antispasmodic, anti-inflammatory, antimicrobial, and expectorant effects [127]. *Pinus sylvestris* L. is used in traditional medicine against asthma, tuberculosis, bronchitis, or other respiratory infections [37,132]. Modifications of abietic and levopimaric acids from *Pinus sylvestris* L. resin led to the development of derivatives that demonstrated their efficacy when tested on NSCLC cell lines [133,134]. Additionally, low toxicity was observed on normal cell lines [133]. Studies assessing the toxicity of *Pinus sylvestris* L. extracts and its bioactive compounds against different LC and normal cell lines are presented in Table 5. Also, some major bioactive compounds from extracts are listed.

### 4.7. Plantago major *L.*

*Plantago major* L. (greater plantain) is an herbaceous species of the Plantaginaceae family. This family consists of about 94 genera and 1900 species. *Plantago major* L. is native to Eurasia. The plant grows in meadows, wastelands, and wet areas. It can reach 20–80 cm height; the stems are short; the leaves are large, ovate to elliptic in shape, with 3/5/7 veins; the surfaces of the leaves are subglabrous or with hair; the inflorescences are in the form of a spike; and the fruit is a capsule [137].

*Plantago major* L. is a medicinal plant. Its biologic activity is attributed to a variety of chemical compounds including flavonoids (luteolin, apigenin, and baicalein), alkaloids, triterpenic acids (ursolic acid and oleanolic acid), phenolic compounds (caffeic acid derivatives), glycosylated iridoids (aucubin and catalpol), fatty acids, polysaccharides, and vitamins [138,139].

The plant possesses immunomodulatory, antiviral, anti-inflammatory, and anticancer activity [140,141]. However, the mechanisms of proliferation inhibition have not been fully elucidated [141]. Around the world, *Plantago major* L. is used to alleviate skin, respiratory, digestive, urinary tract, gynecological, cardiovascular, and other kinds of disorders [38].

Table 6 presents the effects of *Plantago major* L. extracts and bioactive compounds on LC and normal cell lines. Also, some major bioactive compounds from extracts are listed.

Studies reporting the protective effects of *Plantago major* L. extracts on renal toxicity induced by chemotherapy in rats are summarized in Table 7. Ursolic acid extracted from *Plantago major* L. has anti-inflammatory and antioxidant properties. It might have a protective effect against cyclophosphamide-induced hemorrhagic cystitis [27].

### 4.8. Sambucus nigra *L.*

*Sambucus nigra* L. (common elder) is a shrub belonging to the Adoxaceae family (Caprifoliaceae family) [152]. The Caprifoliaceae family consists of about 42 genera and 860 species [153]. The plant is frequently found in Europe, North Africa, Scandinavia, and Great Britain. It grows on forest edges and in flooded plains or shrublands. It can reach up to 10 m in height; the branches are arched and the leaflets are ovate; the flowers are hermaphrodite, white, grouped in top flattened inflorescences; and the fruit is a black drupe [152].

The most used parts of this plant are the berries and the flowers. The berries contain 0.45–1.4% anthocyanins. Due to the anthocyanin content, elderberry extracts are used as natural food dyes with beneficial health effects [154]. The *Sambucus nigra* L. flower dichloromethane extract contains fatty acids (palmitic, stearic, oleic, etc.), carbohydrates (rhamnose and cellobiose), amino acid derivatives (methyl leucinate), phenolic acids (ρ-anisic, ferulic, benzoic, 4-coumaric, and cinnamic acids), tyrosol, flavonoids (naringenin), and monoterpenes (linalool oxide). *Sambucus nigra* L. flower aqueous extracts have numerous phenolic compounds (caffeoylquinic, ferulic, caffeic, and coumaric acids; quercetin; kaempferol; and isorhamnetin) [155].

*Sambucus nigra* L. has antioxidant [156], antiviral [157], anti-inflammatory, and anticancer properties [158]. In traditional medicine, *Sambucus nigra* L. is used for the treatment of skin ailments (burns and wounds; pimples), respiratory disorders (asthma and cough), and eye inflammation [39]. Studies assessing the toxicity of *Sambucus nigra* L. extracts against LC and normal cell lines are presented in Table 8. Also, some major bioactive compounds from extracts are listed.

Aqueous extracts obtained from elderberries activate the proliferation of peripheral blood mononuclear cells, which consist of a mixture of cells involved in the immune response such as lymphocytes, macrophages, and monocytes. By stimulating the proliferation of these cells, elderberry extracts promote immunity in healthy people or those suffering from diseases including cancer [154].

### 4.9. Thymus serpyllum *L.*

*Thymus serpyllum* L. (wild thyme) is a shrub belonging to the Lamiaceae family. It originated from most of Europe. It grows in places with sandy, rocky, or dry soils [162]. Its height can reach 12–13 cm and these plants have thin [163] and long stems, which are woody in the lower parts. The leaves are glabrous and oval with rounded tips; their bases are conical and have trichomes; the central rib is prominent, and the lateral ones are less prominent. They are 4–6 mm long and 2–4 mm wide. Inflorescences of 4–7 cm appear in the upper parts of the stems and the flowers are grouped in spherical or elongated spikes [162].

*Thymus serpyllum* L. is rich in phenolic compounds such as gallic, caffeic, and 4-hydroxybenzoic acids; rutin; naringin; and catechol [164]. Another important constituent of *Thymus serpyllum* L. is its volatile oil, of which thymol, carvacrol, p-cymene, cineol, nerolidol, limonene, geraniol, germacrene, linalool, geranyl acetate, and borneol are the main bioactive compounds [25,129,165,166].

*Thymus serpyllum* L. extracts and volatile oils possess a variety of biological effects, such as antioxidant, antibacterial, and antifungal activities. The extracts exerted spasmolytic, anti-inflammatory, and antitumor properties in a study [167]. The plant is recommended in respiratory and digestive disorders [40]. Despite its cytotoxic effect on LC cell lines, some researchers have reported that *Thuymus serpyllum* L. volatile oil has strong cytotoxic effects on human normal lung fibroblasts (MRC5) [25]. Table 9 presents the effects of *Thymus serpyllum* L. essential oil and bioactive compounds on LC and normal cell lines. Also, some major bioactive compounds from essential oil are listed.

### 4.10. Tussilago farfara *L.*

*Tussilago farfara* L. (coltsfoot) is the only species of the genus *Tussilago,* belonging to the Compositae family [169], also known as the Asteraceae family [170]. It grows in Europe and Asia. The plant is found on the banks of rivers and in crop fields, wastelands, or roadsides. This plant has a height of 5–15 cm; the leaves are large and round and they have a heart shape. Also, these plants have long petioles, radial veins, and wrinkled, toothed leaves. The floral buds are in the shape of long and round rods, 12.5 cm in length and 0.5–1 cm in diameter. The upper part is bent or with a short pedicel. It is covered with numerous bracts similar to fish scales. Floral buds appear earlier, in February–March, before the leaves. The flowers are yellow, similar to dandelion flowers [169].

The leaves of *Tussilago farfara* L. contain amino acids (valine, leucine, threonine, phenylalanine, arginine, alanine, serine, etc.), phenolic acids (caffeic, ferulic, and 3,4-hydroxybenzoic acids), and other acids (ascorbic, malic, and citric) [171]. The hydroethanolic extract from the aerial parts of *Tussilago farfara* L. is rich in phenolic acids (with chlorogenic acid being the most abundant) and flavonoids (with the major compound being hyperoside or myricetin, depending on the collection areas of plant products) [172].

A review published by Chen et al. highlighted the antioxidant, antimicrobial, anti-inflammatory, immunostimulating, antitumor, α-glucosidase-inhibitory, and neuroprotective properties of this plant [169]. *Tussilago farfara* L. has been used in traditional medicine in the treatment of respiratory ailments (asthma, colds, bronchitis, tuberculosis, cough, and phlegm) and skin problems (wounds and burns) [41]. Table 10 presents the effects of *Tussilago farfara* L. extracts on LC cell lines. Also, some major bioactive compounds from extracts are listed.

Nazeam et al. have shown that the polysaccharide precipitate fraction from *Tussilago farfara* L. has a cytotoxic effect on A549 cells while the filtrate obtained after polysaccharide precipitation has a lower cytotoxic effect [175].

Safonova et al. also claimed that polysaccharides from *Tussilago farfara* L. had antitumor effects. In addition, they also reported antimetastatic effects. They showed that *Tussilago farfara* L. polysaccharides decreased the expression of programmed cell death-1 protein (PD-1) and the ligand of programmed cell death-1 protein (PD-L1) in peripheral blood and tumor tissue lymphocytes in vivo in C57BL/6 mice with Lewis LC [176]. Furthermore, some studies demonstrated that polysaccharides from *Tussilago farfara* L. protected the intestinal epithelium in C57BL/6 mice with Lewis lung carcinoma from damaged produced by fluorouracil [177] or polychemotherapy containing cisplatin [178]. Polysaccharides from *Tussilago farfata* L. increased the efficacy of polychemotherapy with cisplatin and paclitaxel and alleviated the neutropenia induced by this therapy [179]. Moreover, polysaccharides protected bone marrow cells and the small-intestinal epithelium against damage induced by polychemotherapy [28].

Chronic obstructive pulmonary disease (COPD) can contribute to the development of LC. *Tussilago farfara* L. is found in the top 10 most used plants in Chinese herbal medicine for treating COPD. In a study, the patients using Chinese herbal medicine to treat COPD had a lower risk of developing LC compared to those using Western therapy. The patients using Chinese herbal medicine along with inhaled corticosteroids had a lower risk of developing LC while those who used short-acting beta-adrenomimetics without Chinese herbal medicine had an increased risk of developing LC [180]. A literature review mentions *Tussilago farfara* L. as an adjuvant to conventional therapy in LC. Even complementary and adjuvant remedies can have side effects or reduce the effects of conventional therapy [181].

## 5. Parameters for Establishing the Cytotoxic Activity of Plant Extracts

Two of the most important parameters that characterize the cytotoxic effect are IC_50_ and selectivity.

### 5.1. IC_50_

According to the American National Cancer Institute, the IC_50_ for plants and plant crude extracts should be lower than 20 μg/mL (or 10 μM) upon 48 or 72 h of incubation. The extracts with IC_50_ lower than 30 μg/mL are promising, and they could be purified. Concentrations below 100 μg/mL could be efficient, and they are of interest for anticancer drug discovery [182]. The IC_50_s reported in the reviewed articles can be found in Table 1, Table 3, Table 4, Table 5 and Table 6, and Table 8, Table 9 and Table 10. These results contribute to the explanation of the cytotoxic activity of the extracts and bioactive compounds from the plants investigated. The potency of the cytotoxic effect varies depending on the plant species, the plant part used for extraction, the type of extract (solvent used and extraction method), the exposure time, and the type of cell line studied. Among the plant species included in this review, *Achillea millefolium* L. demonstrated the strongest anticancer effect, with many of the studied extracts showing IC_50_ values lower than 30 µg/mL across different cell lines [23,43]. Furthermore, bioactive compounds from *Achillea millefolium* L. exhibited a high antiproliferative effect (achillinin A [47]) and cytotoxic effect (casticin [48]). Good-to-moderate cytotoxic effects were observed for extracts of *Hypericum perforatum* L. [73,75], *Sambucus nigra* L. [159,161], and *Thymus serpyllum* L. [25,165]. Additionally, bioactive compounds from *Hypericum perforatum* L., such as hyperforins, demonstrated strong cytotoxic effects [79]. Chlorogenic acid from *Sambucus nigra* L. also showed significant cytotoxicity [159]. However, studies on *Sambucus nigra* L. and *Thymus serpyllum* L. species are limited. Weaker cytotoxic or antiproliferative effects were generally observed for *Mentha longifolia* L. [118,124], *Plantago major* L. (except for ursolic and oleanolic acids, which demonstrated better effects) [139,140], and *Tussilago farfara* L. [19,173,174]. Extracts of *Lythrum salicaria* L. and *Melilotus officinalis* L. are less studied for their cytotoxic effects on lung cancer cell lines, but bioactive compounds found in their composition have proven their effectiveness [99,100,101,102,112,113,114,115].

The IC_50_ value is frequently used in studies to express the toxicity of compounds on cell lines. The literature shows variations in the IC_50_ value obtained when testing the cytotoxicity of a compound on a cell line, although ideally, it should remain consistent. Differences in IC_50_ values are concerning and pose a challenge for translating in vitro results to clinical studies. Factors contributing to the differences in IC_50_ when testing the cytotoxicity of a compound on a cell line include the cell seeding density, the number of cells used for testing, the exposure time, the type of assay, and differences in the units used to express concentrations [183].

These variations are even more likely when testing plant extracts as they are complex mixtures of bioactive compounds. The use of bioactive plant compounds in combinations for cancer therapy is limited due to insufficient knowledge of their pharmacokinetic and pharmacodynamic properties, low bioavailability, or high toxicity at large doses. In some cases, structural modifications or formulation strategies are employed to improve the pharmacodynamic and pharmacokinetic profiles of secondary metabolites, ensuring they reach their target sites of action [184].

### 5.2. Selectivity

A desired characteristic of cytotoxicity is selectivity, so the cytotoxic agent should induce a toxic effect on cancer cells, but it should not affect normal ones [182]. Some of the studies included in this review investigated the cytotoxic effects on both cancerous and normal cell lines. For example, Zhou et al. reported that the cytotoxic effect of casticin is higher on NSCLC cell lines (H460, A549, and H157), with decreased IC_50_ values compared to those found in human embryo lung cells (wi-38) [48]. The hydromethanolic extract of *Achillea millefolium* L. showed an increased cytotoxicity against the NCI-H460 LC cell line compared to the Vero cell line [60]. Hyperoside induced autophagy in A549 cells. This process was not observed in the human bronchial epithelial cell line (Beas-2B) [76]. In contrast, some plant extracts exhibited cytotoxic effects both on cancerous and on normal cells. An extract of *Hypericum perforatum* L. produced by the Natural Products company and dissolved in DMSO exhibited cytotoxic effects on both A549 and healthy human lung fibroblast cells (CCD-34Lu) [73]. The cytotoxic effect of *Mentha longifolia* L. essential oil was reported on the A549 NSCLC cell line [123], but its toxicity was very low on the normal Homo sapiens human lung fibroblast cell line wi-38 [118]. Moreover, no cytotoxic effect was observed on normal vein endothelial cells (HUVECs) for this volatile oil [123]. Rangam et al. reported the cytotoxic effects of nanocomposites with a *Pinus sylvestris* L. aqueous extract on the A549 cell line, but these nanocomposites were biocompatible with normal human embryonic kidney cells (HEK293) [136]. The aqueous extract of *Pinus sylvestris* L. had low cytotoxicity against normal human lung fibroblasts (IMR90) [135]. Some propargylated methyl dihydroquinopimarates demonstrated low cytotoxicity against HEK293 and did not affect red blood cells [133]. A lack of toxicity was reported for the *Plantago major* L. ethanol extract on Beas-2B [142] and PMS on Chinese hamster ovary cells [143]. The cytotoxicity of *Thymus serpyllum* L. volatile oils was reported on both A549 cells and human normal lung fibroblasts [25].

## 6. Mechanisms of Action Involved in the Cytotoxic Effect

In general, the cytotoxic effects of plant extracts and their bioactive compounds are analyzed by 3-(4,5-dimethylthiazol-2-yl)-2,5-diphenyltetrazolium bromide (MTT) assay. The MTT assay is a colorimetric test for assessing cell viability. The MTT assay is a rapid, non-radioactive, economical, and accurate assay that is widely used in the laboratory, but it depends on cell metabolism and interferes with some substances and NPs [185].

Furthermore, some studies included other tests to identify the mechanisms of action involved in cytotoxicity. Most of the studies included in this review reported that the cytotoxic effects of plant extracts or their bioactive compounds were produced by the following main mechanisms: cell death induction by apoptosis, necrosis, and autophagy; cell cycle arrest; signaling pathway modulation (of the phosphoinositide 3-kinase (PI3K)/protein kinase B (Akt) pathway and mitogen-activated protein kinase (MAPK) pathway); antiangiogenesis; the inhibition of migration; invasion and metastasis; and targeting inflammation.

### 6.1. Induction of Cell Death

#### 6.1.1. Apoptosis

Many of the studies included in this review explained the cytotoxic and antiproliferative effects of plant extracts and bioactive compounds through apoptosis [16,17,18,19,20,29,30,45,48,61,62,76,77,174]. Apoptosis is the most studied process of cell death. It consists of two pathways (intrinsic and extrinsic) converging to a final pathway (the execution pathway). The intrinsic pathway involves mitochondria and is also called the mitochondrial pathway. The effector proteins B-cell lymphoma 2 (Bcl-2), homogenous antagonist/killer (Bak), and Bcl-2 associated x protein (Bax) are inserted into the external mitochondrial membrane. Then, cytochrome c is released from the intermembrane space into the cytosol. Here, apoptosomes are formed of cytochrome c, apoptotic protease-activating factor-1 (APAF-1), and procaspase-9, which is activated as caspase-9. Caspase-3/-6/-7 are then activated, and apoptosis occurs. The Bcl-2 and Bcl-2-like protein 1 (Bcl-xL) proteins are antiapoptotic proteins that inhibit the release of cytochrome c. The activity of antiapoptotic proteins can be inhibited by the proteins phorbol-12-myristate-13-acetate-induced protein 1 (Noxa), p53 up-regulated modulator of apoptosis (Puma), Bcl-2 associated agonist of cell death (Bad), Bcl-2-like protein 4/Bcl-2 interacting mediator of cell death (Bim), and BH3 interacting-domain death agonist (Bid). Noxa and Puma are stimulated by the p53 protein. The inhibitions of the PI3K/Akt pathway and the cell cycle block the transmission of survival stimuli and can regulate Bad protein activity. The C-Jun N-terminal kinase (JNK) pathway can stimulate Bax. Extrinsic apoptosis occurs through the stimulation of death receptors on the surface of the cell membrane by ligands that are found outside the cell such as tumor necrosis factor-α (TNF-α), Fas ligand (FasL), or TNF-related apoptosis-inducing ligand (TRAIL). Next, caspase-8 and -10 are activated, which activate caspase-3, -6, and -7, leading to apoptosis. The two pathways are interconnected by the Bid protein and caspase-3, -6, and-7. Poly-(ADP-ribose) polymerase (PARP) is involved in the final stage of apoptosis along with other factors that cause DNA fragmentation. X-linked inhibitor of apoptosis protein (XIAP) inhibits apoptosis by down-regulating caspases [186,187].

**Figure 2:**
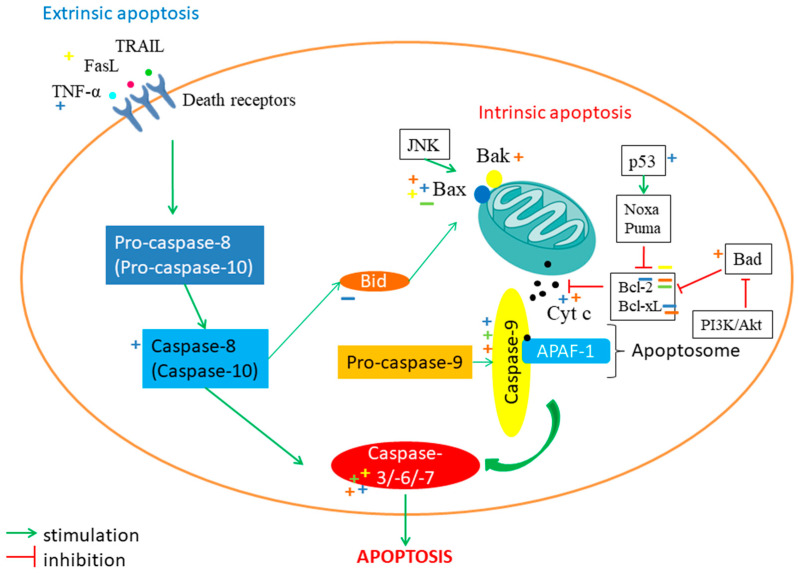
The sites of action of different medicinal plants on the apoptosis pathway (“+”/“−”: up-regulation/down-regulation produced by plant extracts and their bioactive compounds; “**+**”/“**−**”represents *Achillea millefolium* L.; “**+**”/“**−**” represents *Hypericum perforatum* L.; “**+**”/“**−**” represents *Plantago major* L. and “**+**”/“**−**” represents *Tussilago farfara* L.) (adapted after [18,19,29,30,31,45,48,61,62,77,78,186,187]).

*Achillea millefolium* L. extracts and bioactive compounds induce apoptosis by activating the intrinsic pathway. It was reported that p53, Bax, and caspase-3 are up-regulated by millifolide A in A549 cells and Achillea millefolium A in NCI-H292 cells. Moreover, in studies, millifolide A increased the expression of pG53-Luc and pGBax-Luc in A549 cells and Achillea millefolium A increased the expression of caspase-3, -8, and -12 and GRP78 in NCI-H292 cells [18,62]. The hydroethanolic extract rich in phenolic acids and flavonoids induced p53 and p21 expression but inhibited the expression of pro-caspase-3 and PARP in NCI-H460 cells [29]. Casticin induced p21 and inhibited Bcl-2 in H1299 cells [61]. Casticin also stimulated caspase-3, -8, and -9 in H460, A549, and H157 cells. Moreover, in H157 cells, casticin increased the release of cytochrome c, induced Bax expression, and down-regulated XIAP, Bcl-XL, and Bid [48]. Isovitexin associated with cisplatin therapy induced apoptosis in A549 and H1975 cells by stimulating the extrinsic pathway via increasing TNF-α [45]. Hyperoside, a bioactive compound from *Hypericum perforatum* L., induced the apoptosis of A549 and H1975 cells by modulating the expression of proteins. Among the mechanisms, the up-regulation of caspase-3 and -9, Bax, Bad, Bak, and PARP; increasing the release of cytochrome c; and the down-regulation of Bcl-2, Bcl-xL, and cellular FLICE inhibitory proteins (c-FLIPs) were involved [20,30,76,77,78]. Inducing apoptosis by chitosan NPs with hypericin on the A549 cell line [16] and hyperforin on the CL1-5F4 cell line was also reported [17]. Plantamajoside, a bioactive compound from *Plantago major* L., influenced the expression of the proteins involved in intrinsic apoptosis. It down-regulated Bax and Bcl-2 and increased the expression of cleaved caspase-3 and -9 and the Bax/Bcl-2 ratio [31]. Song et al. reported that a sesquiterpenoid from *Tussilago farfara* L. showed an increased apoptotic rate of A549 cells compared to doxorubicin [174]. TFPB1, a polysaccharide from *Tussilago farfara* L., increased apoptosis in a dose-dependent way. It modulated both intrinsic and extrinsic pathways by increasing the expression of caspase-3 and Bax, decreasing the expression of Bcl-2, and up-regulating Fas and FasL [19]. Figure 2 illustrates the sites of action of different medicinal plants on the apoptosis pathway.

Moreover, apoptosis can be a cause of excess intracellular ROS that causes the destruction of proteins, lipids, cell organelles, and nucleic acids [188]. ROS play a dual role in cancer. Low and moderate levels drive migration, invasion, and angiogenesis, while high levels activate cell death [189]. Taşkonak et al. studied the role of chitosan NPs with hypericin in LC photodynamic therapy. An increase in ROS was observed in the group treated with chitosan NPs with 600 nM hypericin compared to the control group and the one treated only with hypericin [16]. PMS increased the oxidative stress in the metastatic 95D cell line [31]. *Thymus serpyllum* L. essential oil was reported to decrease ROS production in the MRC-5 cell line [25].

#### 6.1.2. Necrosis

**Figure 3:**
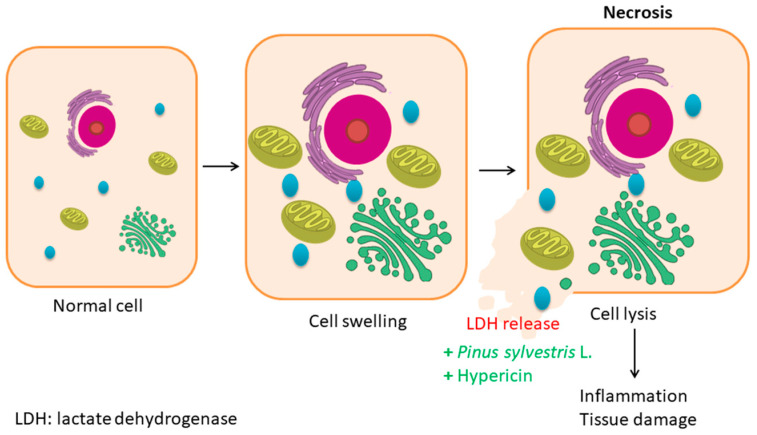
Necrotic cell death induced by plant extracts and their bioactive compounds (adapted after [16,136,190,191].

Necrosis (Figure 3) is another type of cell death. It is produced after the exposure of cells to external factors such as radiation, chemicals, hypoxia, etc., which cause swelling in the cell, resulting in the rupture of the cell membrane and release of cell contents into the surrounding area. Then, inflammation and tissue destruction occur [190]. Lactate dehydrogenase (LDH) is an enzyme that is released from the cell when the cell membrane is disturbed. Measuring the amount of LDH released from cells is a useful test for detecting necrosis [191]. Some extracts or bioactive compounds act by increasing LDH release. Rangam et al. studied the release of LDH from A549 cells treated with nanocomposites with a *Pinus sylvestris* L. aqueous extract. They observed a higher LDH release at lower concentrations, which meant that the cells did not grow and divide when treated at higher concentrations [136]. Chitosan NPs with 600 nM hypericin showed the highest release of LDH, leading to increased necrotic cell death [16].

#### 6.1.3. Autophagy

Autophagy is a non-apoptotic type of programmed cell death [76] whereas in cancer cells, autophagy would have a tumor suppressor effect. Autophagy involves the sequestration of a portion of the cytoplasm into autophagosomes. These fuse with lysosomes to form autolysosomes. The content of the autolysosomes is degraded and spilled back into the cytoplasm, where the components are recycled by the cell. There are three types of autophagy mechanisms: macro-, micro-, and chaperone-mediated. Among them, macroautophagy is the most studied [192]. It has been reported that hyperoside induces autophagy in A549 cells [76].

### 6.2. Cell Cycle Arrest

The cell cycle, responsible for cell division, ensures the transmission of genetic information from an existing cell generation to the next generation [193]. Cell division is a controlled and ordered process. It consists of numerous checkpoints that evaluate the growth signals from the exterior of the cell, the size of the cell, and the integrity of the DNA [194]. The cell cycle has five phases. The gap 1 (G1) phase is the stage in which the cell prepares for DNA synthesis. Mitogenic factors and growth stimuli from outside the cell appear. The synthesis (S) phase is represented by DNA replication. The gap 2 (G2) phase is the stage in which the cell prepares for mitosis (the M phase). During mitosis, all cell components are divided between two identical daughter cells. Bipolar mitotic spindle formation, chromosome segregation, and cell division occur. In the absence of mitogenic signals or growth factors, the cell exits the cell cycle and enters the resting phase (G0), in which no division occurs [193,194,195].

Cell cycle regulation is achieved by catalytic complexes of cyclins and cyclin-dependent kinases (CDKs). In the G1 phase, cyclin D forms complexes with CDK4/6; the phosphorylation and activation of CDK occur. They phosphorylate retinoblastoma (RB) proteins. The increased expression of the transcription factor (E2F) and formation of cyclins E occur. Towards the end of the G1 phase, cyclin E complexes with CDK2, leading to the phosphorylation of RB proteins. At the transition between the G1 and S phases, cyclin A expression increases. In the S phase, the cyclin A/CDK2 complex activates DNA polymerase α and chromosome replication occurs. In the latter part of the S phase, cyclin A is associated with CDK1. The G2 phase is regulated by cyclin A/CDK1 and cyclin B/CDK1 complexes. At G2/M transition, the cyclin A/CDK1 complex is activated. Next, cyclin A is degraded and cyclin B1/CDK1 complex is activated. Then, cyclin B1 is degraded by the anaphase-promoting complex or cyclosome. Other regulators of mitosis are Aurora and the polo-like kinase [61,193,194]. Cellular damage caused by oxidative stress or DNA damage by chemical agents can lead to cell cycle arrest. Stressors induce the expression of the tumor suppressor protein p53, which acts in the G1 and G2/M phases. p53 decreases the expression of cyclins and CDKs and increases the expression of CDK-inhibitory protein (p21). p53 can cause apoptosis by inhibiting Bcl-2 [61].

**Figure 4:**
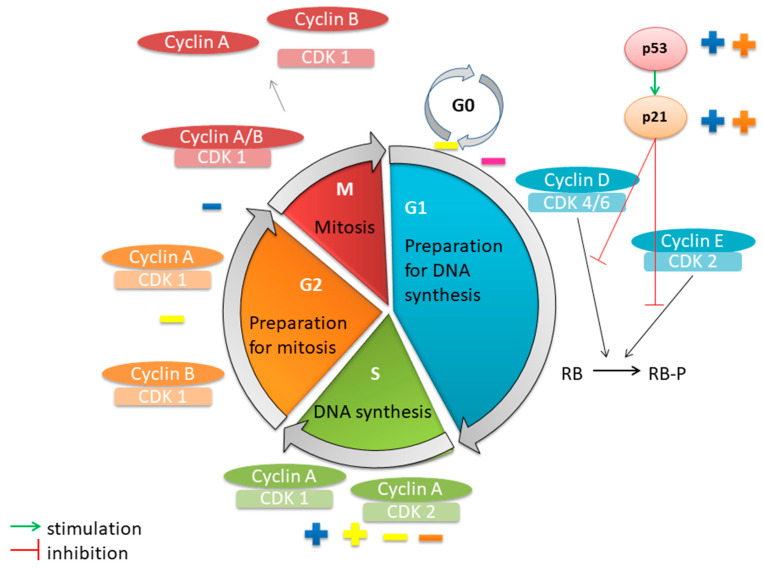
The influencing of cell cycles by plant extracts and their bioactive compounds (“+”/“−”: stimulation/inhibition produced by plant extracts and their bioactive compounds; “**+**”/“**−**”represents *Achillea millefolium* L.; “**+**”/“**−**” represents *Hypericum perforatum* L.; “**−**” represents *Thymus serpyllum* L.; “**+**”/“**−**” represents *Tussilago farfara* L.) (adapted after [19,29,77,174,193,194,195]).

Studies have reported that plant extracts and their bioactive compounds possess antitumor activity by influencing the cell cycle in different phases. Thus, Pereira et al. observed that an *Achillea millefolium* L. hydroethanolic extract at 100 μg/mL decreased the percentage of cells in the G2/M phase, increased the percentage of cells in the S phase, and stimulated the expression of p53 and p21 in the NCI-H460 cell line [29]. Casticin from *Achillea millefolium* L. also triggered cell cycle arrest in the G2/M phase after 24 h of exposure to a concentration equal to IC_50_. Furthermore, it decreased the expression of cyclin A and slightly increased the expression of cyclin B and p21 in the H1299 cell line [61]. Hyperoside from *Hypericum perforatum* L. exhibited S-phase cell cycle arrest in the A549 cell line. It inhibited the expression of cyclin D and CDK1, but increased the expression of p53, p27, and p21 [77]. Thymol, a major compound of the *Thymus serpyllum* L. volatile oil, caused cell cycle arrest in the G1 phase [25]. TFPB1 from *Tussilago farfara* L. influenced the cell cycle in different phases. In the G0–G1 phases, there was a dose-dependent decrease in the number of cells, while in the S phase, an increase in the percentage of cells was observed [19]. Another compound from *Tussilago farfara* L. ethanol extract induced cell cycle arrest in the G2 and S phases of the A549 cell line [174]. *Tussilago farfara* L. ether petroleum extract decreased the expression of Aurora-A in the LLC cell line [173]. Figure 4 illustrates the influencing of cell cycles by plant extracts and their bioactive compounds.

### 6.3. Modulation of Signaling Pathways

#### 6.3.1. Inhibition of the PI3K/Akt Pathway

The excessive activation of the PI3K/Akt pathway can occur in LC and is associated with a poor prognosis. Different stimuli from outside the cell, such as cytokines, growth factors, or hormones, can bind to the receptor tyrosine kinase with the activation of the PI3K/Akt pathway. Thus, the conversion of phosphatidylinositol-(4,5)-bisphosphate (PIP2) to phosphatidylinositol-(3,4,5)-triphosphate (PIP3) occurs. Through the pleckstrin homology domain, PIP3 recruits phosphatidylinositol-dependent kinase-1 (PDK1) to the cell membrane. Next, threonine 308 is formed by the phosphorylation and activation of the serine/threonine kinase Akt by PDK1. The full activation of Akt occurs when serine 473 is phosphorylated by the mammalian target of rapamycin complex 2 (mTORC2). Following the full activation of Akt, cell proliferation, differentiation, survival, metabolism, and metastasis are stimulated [102]. Hyperoside from *Hypericum perforatum* L., PMS from *Plantago major* L., and TFPB1 from *Tussilago farfara* L. influence this pathway by inhibiting the phosphorylation of Akt [19,31,76,77].

#### 6.3.2. Modulation of MAPK Pathway

The MAPK pathway ensures the transformation of signals from outside the cell into responses at the cellular level. There are three families of MAPKs in mammalian cells, each exhibiting certain responses, namely classic MAPK or extracellular-signal-regulated kinase (ERK), which is involved in cell proliferation, differentiation, and development; C-Jun N-terminal kinase/stress-activated protein kinase (JNK/SAPK), which is involved in proliferation, differentiation, cell development, inflammation, apoptosis, and stress response; and p38 kinase, which is involved in proliferation, differentiation, and apoptosis [196]. Yang et al. investigated the effect of hyperoside on A549 cells. Using Western blot tests, they observed that hyperoside (10, 50, and 100 μmol/L) increased the degree of phosphorylation of JNK and p38 kinase [30]. Additionally, Fu et al., using the same test, reported that hyperoside (0.5, 1, and 2 mmol/L) stimulated the phosphorylation of ERK1/2 [76]. PMS (50, 100, and 200 μg/mL), a bioactive compound from *Plantago major* L., inhibited the phosphorylation of p38 protein in a metastatic 95D cell line [31].

### 6.4. Inhibition of Migration, Invasion, and Metastasis

Migration, invasion, and metastasis are based on the mechanical degradation of the extracellular matrix by proteinases associated with tumors, such as MMPs. These proteinases are targets for anticancer therapy. By inhibiting them, tumor dissemination and metastasis are prevented. Synthetic drugs that inhibit MMPs have been developed, but they have shown increased toxicity in clinical trials. Several compounds available in vegetal products have been observed to inhibit MMPs without causing toxic effects [80]. In studies, PMS decreased lung metastasis by inhibiting MMP2/9 [143]. Hyperoside decreased the migration and invasion of A549 cells by inhibiting MMP2/7/9 and H1975 cells did so by inhibiting MMP2/7 [77,78]. Also, hyperoside decreased the expression of the vascular endothelial growth factor (VEGF). VEGF expression is closely related to LC progression. An increased expression is associated with a poor prognosis for patients suffering from this disease [78]. A polyphenolic derivative extracted from *Hypericum perforatum* L., hyperforin, inhibits matrix proteinases and cancer cell proliferation [197].

Vargová et al. reported that hypericin competitively inhibits the breast carcinoma resistance protein (BCRP) and decreases the side population of A549 cancer cells [198]. Another study highlighted the dual role of hypericin regarding side populations of cancer cells. From one point of view, hypericin is, at the same time, a substrate and an inhibitor of BCRP, and it can modulate the side population and hypoxia-induced factors. BCRP induces drug resistance by drug efflux. Hypoxia-induced factors are related to the poor prognosis of the disease. Hypericin decreases the side population through the accumulation in hypoxic cancer cells, the degradation of hypoxia-induced factors, the decrease in the efflux given by the BCRP, and the decrease in hypoxia. On the other hand, hypericin stimulates the migration of these cell populations [199].

### 6.5. Antiangiogenesis

Angiogenesis represents the formation of new blood vessels from existing blood vessels. This process involves the degradation of glycoproteins from basal membranes and other components from the extracellular matrix that surround blood vessels; activation, migration, and proliferation of endothelial cells; transformation of endothelial cells in capillary tubes; and formation of new basal membranes [200]. Hyperoside from *Hypericum perforatum* L. decreased the number of blood vessels in a study [77]. PMS from *Plantago major* L. inhibited angiogenesis as a result of MMP2 inhibition and the decreasing of lung metastases [143].

### 6.6. Targeting Inflammation

Chronic inflammation contributes to the initiation and progression of several diseases including LC [201]. Elements with various roles in inflammation are present in the tumor microenvironment, promoting tumor proliferation, metastasis, invasion, and angiogenesis [202]. An increase in the expression of TNF-α [203] and interleukins IL-1β, IL-6, and IL-8 [204] has been observed in patients with LC. TNF-α is an inflammatory cytokine that activates the production of IL-1β and IL-6 [203]. TNF cytokines stimulate the Nuclear Factor Kappa B (NF-kB) transcription factor, which plays a central role in cell proliferation and differentiation [205]. Some medicinal plants and their bioactive compounds possess beneficial anti-inflammatory effects in fighting LC. In a study, hyperforin inhibited the NF-kB activation in CL1-5-F4, which decreased antiapoptotic proteins and cell invasion [17]. Hyperoside down-regulated NF-kB in A549 cells [77,78], but also induced anti-inflammatory effects by down-regulating TNF-α and the interleukins IL-6, IL-1β, and IL-18 [78]. Cisplatin decreased the immune cell function in mice with induced lung tumors while isovitexin and the concomitant administration of isovitexin and cisplatin increased immune cells’ (T cells, B cells, cytotoxic T lymphocytes, and natural killer cells) activity and stimulated the production of IL-2 and TNF-α [45].

Our research aimed to provide an overview of the cytotoxic effects and protective potential against chemotherapy-induced toxicity of the ten plants from the spontaneous flora. However, we encountered several gaps in the literature, which represent some limitations of our review. Variations in the preparations of extracts, tested dosages, cell lines used, types of extracts, and different expressions of IC_50_ values make it difficult to compare results. Although many studies have investigated the beneficial effects in vitro, few have reported the mechanisms of action, and the validation of results through clinical trials is lacking. As plant extracts are mixtures of bioactive compounds, it is difficult to determine exactly which component is responsible for a beneficial effect. The lack of standardization in the composition of the extracts is an impediment to translating the findings into clinical practice. Future studies could focus on assessing the effects of individual compounds, establishing doses, and evaluating drug–drug interactions.

## 7. Conclusions

This review has highlighted not only the cytotoxic potential on LC cell lines but also the protective effect against chemotherapy-induced toxicity of ten medicinal plants from spontaneous flora. Plant extracts, volatile oils, and bioactive compounds have been included. The triggered mechanisms of action have been presented, explaining the cytotoxic effects based on the induction of apoptosis; interference with cell cycle regulation; the inhibition of angiogenesis; migration, invasion, and metastasis; blocking the PI3K/Akt pathway; ROS production; LDH release; autophagy; inflammation; and the modulation of the MAPK pathway. Due to their varied constitutions, natural products can exert both cytotoxic and protective effects. Phenolic compounds like hypericin, hyperforin, hyperoside, PMS, etc. were reported to induce cytotoxic effects. Other compounds such as isovitexin from *Achillea millefolium* L. and polysaccharides from *Tussilago farfara* L. feature synergistic effects in association with chemotherapy. Furthermore, polyphenols, flavonoids, tannins, and saponins could have protective effects against the toxicity induced by chemotherapy. For species such as *Melilotus officinalis* L. and *Lythrum salicaria* L., there have been no in vitro studies demonstrating cytotoxicity. The phytonutrients mentioned above, based on the reported results, may play a crucial role in the complementary treatment of lung cancer or potentially contribute to the development of new anticancer drugs. Achillinin A and casticin may be potential candidates for future studies. However, for all the plant species analyzed, the in vivo studies and clinical trials have been few, and these are necessary for the in-depth evaluation of such species to evaluate the efficacy and safety of these products.

## Figures and Tables

**Table 1:** Effects of *Achillea millefolium* L. on LC and normal cell lines.

Plant Origin	Plant Organ	Extract Type	Cell Lines	Bioactive Compounds	Bioactive Compound Concentrations	Effects	Reference
Iran	herb	DMSO ext.	SKLC6	-	-	Cytotoxic effects at IC_50_ = 24.106 µg/mL (ext.) and IC_50_ = 1.633 μM (ctrl 1).	[23]
Portugal	wild inflorescences and upper leaves; commercial herb	MeOH ext. infusion decoction	NCI-H460	phenolic acid: cis/trans 3,5-diCQA flavonoids: O-acetylhexoside of luteolin O-acetylhexoside of apigenin kaempferol, quercetin, and isorhamnetin glycoside derivatives	wild sample (35.73/26.02 mg/g MeOH ext., 28.05/19.96 mg/g infusion, 7.40/6.85 mg/g decoction), commercial sample (25.30/10.50 mg/g MeOH ext., 28.45/13.46 mg/g infusion, 27.83/11.98 mg/g decoction) wild sample (6.21 mg/g MeOH ext., 5.32 mg/g infusion, 2.80 mg/g decoction), commercial sample (6.49 mg/g MeOH ext., 7.22 mg/g infusion, 6.80 mg/g decoction) wild sample (5.45 mg/g MeOH ext., 5.89 mg/g infusion, 2.72 mg/g decoction), wild sample (9.85 mg/g MeOH, 12.12 mg/g infusion, 9.47 mg/g decoction)	Antitumor effects at IC_50_ = 24.64 ± 0.80 μg/mL (commercial herb MeOH ext.), IC_50_ = 29.17 ± 4.12 μg/mL (wild inflorescences and upper leaf infusion), IC_50_ = 54.24 ± 0.46 μg/mL (wild inflorescences and upper leaves’ MeOH ext.), IC_50_ = 55.71 ± 0.04 μg/mL (commercial herb decoction), IC_50_ = 56.26 ± 1.15 μg/mL (commercial herb infusion), and IC_50_ = 56.24 ± 3.09 μg/mL (wild inflorescences and upper leaf decoction). Toxicity against PLP2 at higher IC_50._	[44]
Portugal	-	H_2_O/EtOH ext.	NCI-H460	phenolic acids: cis/trans 3,5-diCQA 5-CQA Flavonoids: O-acetylhexoside of luteolin and apigenin apigenin, luteolin, quercetin, kaempferol, and isorhamnetin glycoside derivatives	7.55/5.51 mg/g 5.12 mg/g 1.40 mg/g 1.14 mg/g	Cell cycle inhibition and apoptosis (75 and 100 μg/mL) Antiproliferative effects at IC_50_ = 187.3 ± 25.7 μg/mL ↓ viable cells by 28% (75 μg/mL) and by 55% (100 μg/mL).	[29]
Soria Natural and Pinisan	-	MeOH/H_2_O ext. (60:40 *v*/*v*)	NCI-H460 Vero	3-CQA	18.85 mg/mL	Cytotoxic effects at IC_50_ = 125 µg/mL (NCI-H460 cells).	[60]
Egypt	aerial part	PE, EA, MeOH, and hot H_2_O ext.	A549	EA ext.: apigenin, p-coumaric, chlorogenic acids H_2_O ext.: rosmarinic acid	1.03 mg/g, 2.21 mg/g, 1.45 mg/g 2.49 mg/g	Cytotoxic effects at IC_50_ = 15.84 ± 0.27 μg/mL (PE ext.), IC_50_ = 1.05 ± 0.41 μg/mL (EA ext.), IC_50_ = 1.15 ± 0.56 μg/mL (MeOH ext.), and IC_50_ = 5.60 ± 0.37 μg/mL (H_2_O ext.).	[43]
purchased from Turkey	-	NPs with H_2_O ext.	A549	-	-	Apoptotic effects at IC_50_ = 46.47 μg/mL.	[52]
-	flowers	-	A549 QG-90	iso-seco-tanapartholide; arteludooicinolide A; millifolide A	-	Millifolide A: ↓ A549 and ↑ QG cell viability compared to ctrl 2 Iso-seco-tanapartholide and arteludooicinolide A: low activity Millifolide A and ctrl 2: apoptosis and proliferation inhibition Millifolide A and a caspase inhibitor: proliferation inhibition of A549 from 70.14% to 76.20% (1 μmol/L), 30.41% to 48.09% (10 μmol/L), and 7.37% to 76.20% (100 μmol/L).	[18]
purchased from Canada	flowers	MeOH ext.	QG-90 RERF-LC-kj A549 QG-56 PC-6	2β,3β-epoxy-1α,4β,10α-trihydroxyguai-11(13)-en-12,6α-olide (Achillinin A)	-	Achillinin A: higher antiproliferative than ctrl 2 on some cell lines (IC_50_ = 0.31 μmol/L vs. 31.52 μmol/L, QG-90; IC_50_ = 5.8 μmol/L vs. 11.95 μmol/L, A549; IC_50_ = 10 μmol/L vs. 87.59 μmol/L, RERF-LC-kj) , but lower on others (IC_50_ = 30 μmol/L vs. 27.68 μmol/L, QG-56; IC_50_ = 45 μmol/L vs. 38.35 μmol/L, PC-6).	[47]
-	-	-	A549 H1975	Isovitexin	-	Combined therapy with isovitexin and cisplatin: cytotoxicity by apoptosis The association of isovitexin and cisplatin inhibited the tumor growth in mice more than cisplatin alone. Isovitexin reduced the hepatotoxicity and nephrotoxicity of cisplatin in mice. Isovitexin inhibited glucose metabolism in LC cells.	[45]
-	-	mixture of acetonitrile and H_2_O ext.	H1299	Casticin	-	Cell cycle changes and apoptosis.	[61]
-	-	-	H460 A549 H157 WI-38	Casticin	-	Cytotoxic effects at IC_50_ = 2.3 ± 0.2 μmol/L (H460), IC_50_ = 3.2 ± 0.4 μmol/L (A549), IC_50_ = 1.8 ± 0.1 μmol/L (H157), and IC_50_ = 86.7 ± 5.6 μmol/L (WI-38 cells). These values were lower than those obtained for ctrl 2, except WI-38 cells. Casticin showed cytotoxic effects by apoptosis. The use of caspase inhibitors blocked or reduced the apoptosis induced by casticin. These findings mean that apoptosis is produced only if caspases are activated.	[48]
-	-	-	NCI-H292	3β-acetoxy-1β,4α-dihydroxy-11αH-eudesman-12,6α-olide (Achillea millefolium A)	-	Apoptosis rates of 15.31% (1 μmol/L) and 17.84% (10 μmol/L). In situ end labeling test: 13.89% positive cells after 12 h (10 μmol/L), and 28.37% after 24 h, compared to the blank control (4.17%).	[62]

DMSO: dimethyl sulfoxide, ext.: extract, -: not specified, IC_50_: half-maximal inhibitory concentration, ctrl 1: doxorubicin, MeOH: methanol, 3,5-diCQA: 3,5-O-dicaffeoylquinic acid, H_2_O: water, EtOH: ethanol, 5-CQA: 5-O-caffeoylquinic acid, PLP2: normal liver primary cells, ↓: decreased, 3-CQA: 3-O-caffeoylquinic acid, PE: petroleum ether, EA: ethyl acetate, NPs: nanoparticles, ↑: increased, ctrl 2: cisplatin, and LC: lung cancer.

**Table 2:** Protective effects of *Achillea millefolium* L. extracts against toxicity induced by chemotherapy in rats.

Protective Extract, Posology	Incriminated Compound	Toxic Effect	Reference
*Achillea millefolium* L. hydroalcoholic extract from inflorescences (200, 400 mg/kg) 14 consecutive days	Cisplatin	Ocular toxicity	[64]
*Achillea millefolium* L. ethanol extract (250 mg/kg) 10 days	Cisplatin	Acute vascular injuries of vital organs	[26]
*Achillea millefolium* L. aqueous extract from inflorescences (1.2 g/kg/day) 4 weeks, 4 h after cyclophosphamide	Cyclophosphamide	Reproductive toxicity	[65]
*Achillea millefolium* L. hydroalcoholic extract from flowering parts (200, 400 mg/kg) 14 consecutive days	Paclitaxel	Reproductive toxicity	[66]

**Table 3:** Effects of *Hypericum perforatum* L. on LC and normal cell lines.

Plant Origin	Plant Organ	Extract Type	Cell Lines	Bioactive Compounds	Bioactive Compound Concentrations	Effects	Reference
-	-	NPRO Natural Products company plant ext. dissolved in DMSO	A549 CCD-34Lu	-	-	Cytotoxic effects at IC_50_ = 18.78 ± 1.75 μg/mL (A549) and IC_50_ = 13.58 ± 1.17 μg/mL (CCD-34Lu).	[73]
China	whole plant	MeOH ext.	A549	Hypercohin G	-	Weak cytotoxic activity.	[74]
Turkey	branch-body; flowers	MeOH, EA and hexane ext.	A549 MRC-5	different components depending on the type of extract	-	Flower hexane ext. was cytotoxic at IC_50_ = 20.21 ± 3.52 µg/mL; EA ext. was cytotoxic at IC_50_ = 23.51 ± 1.10 µg/mL. Branch-body hexane ext. was cytotoxic at IC_50_ = 30.35 ± 0.11 µg/mL; EA ext. was cytotoxic at IC_50_ = 55.44 ± 1.70 µg/mL; flower MeOH ext. was cytotoxic at IC_50_ = 93.22 ± 12.28 µg/mL. Branch-body MeOH ext. was cytotoxic at IC_50_ > 200 µg/mL; the cytotoxic effects on the MRC-5 cell line varied with IC_50_ values between 8.13 and 200 µg/mL; or more than 200 µg/mL.	[75]
-	-	peptide extracts	A549 H1299	Peptides	-	No changes in proliferation and no antitumor effect.	[46]
-	-	-	A549	Chitosan NPs with hypericin	-	↑cytotoxic effect of hypericin. ↓ cell viability to 56% after 48 h of incubation.	[16]
Syria	aerial parts	NPs with H_2_O ext.	A549	Phenolic compounds	-	Cytotoxic effect at IC_50_ = 6.08 μg/mL after 24 h of exposure. Concentrations between 11 and 20 μg/mL; ↓ viability by more than 90% after 24 h of exposure.	[24]
-	-	-	CL1-5-F4	Hyperforin	-	↓cell viability.	[17]
-	-	-	PC-9 NCI-H1975 (NSCLC with T790M mutations).	Hyperoside	-	Antiproliferative effect with IC_50_ = 104.1 μM (for 24 h), 87.4 μM (for 48 h), and 70.6 μM (for 72 h) on T790M-positive NSCLC cells (H1975 cells). The growth of the xenografts of T790M-positive NSCLC cell line was inhibited by hyperoside.	[20]
-	-	-	A549	Hyperoside	-	↓cell viability.	[30]
-	-	-	A549 Beas-2B	Hyperoside	-	↓A549 cell viability.	[76]
-	-	-	A549 H1975	Hyperoside	-	Antiproliferative effect on both cell lines. Slight ↑ proliferation (after 12 h) but ↓ viability (after 24 h). Antiapoptotic effect on A549 cell line. Inhibition of cell migration and invasion. ↓ tumor volume, weight, and growth.	[77]
-	-	-	A549	Hyperoside	-	Antiproliferative effect ↓ migration and invasion. Induced apoptosis. Anti-inflammatory effect. ↓ volume and weight of the tumor.	[78]
China	stems and leaves	complex procedure to isolate the bioactive compounds	A549 Beas-2B	hyperforatin A (1), 32-epi-hyperforatin A (2), hyperforatins B−D (3−5), 15-epihyperforatin D (6), hyperforatin E (7), 32-epi-hyperforatin E (8), hyperforatins F−I (9−12), 15-epi-hyperforatin I (13), and hyperforatins J−K (14−15)	-	Cytotoxic effect on A549 cell line with IC_50_s between 13.63 and > 20 μM. IC_50_s for cytotoxic effect on Beas-2B cells varied between 6.38 and 19.71 μM.	[79]

-: not specified, ext.: extract, DMSO: dimethyl sulfoxide, IC_50_: half-maximal inhibitory concentration, MeOH: methanol, EA: ethyl acetate, NPs: nanoparticles, ↑: increased, ↓: decreased, and H_2_O: water.

**Table 4:** Effects of *Mentha longifolia* L. on LC and normal cell lines.

Plant Origin	Plant Organ	Extract Type	Cell Lines	Bioactive Compounds	Bioactive Compound Concentrations	Effects	Reference
India	whole plant	MeOH and H_2_O ext.	A549 NCI-H322	-	-	Antiproliferative effect. MeOH ext. (100 μg/mL): 58% growth inhibition (A549) (less than ctrl 1), and 75% growth inhibition (NCI-H322) (better than ctrl 1, which presented an inhibition of growth of 52%). H_2_O ext. (100 μg/mL): 56% inhibition of growth (A549 cell line) and 31% (NCI-H322) (lower than ctrl 1).	[22]
Iraq	aerial parts	MeOH and CH_2_Cl_2_ ext.	A549	Cinnamic, coumaric, chlorogenic, gallic, benzoic, hydroxybenzoic acids, and catechin	98.17 ppm, 10.01 ppm, 7.3 ppm, 0.64 ppm, 57.05 ppm, 26.27 ppm, 48.22 ppm	MeOH ext.: weak antiproliferative effect. CH_2_Cl_2_ ext.: antiproliferative effect at higher concentrations. The maximum antiproliferative effect was at 200 μg/mL.	[117]
Iran	aerial parts	EO	A549 HUVEC	Carvone Limonene	64.7–72% 7.2–12.9%	Cytotoxic effect at IC_50_ = 48 ± 1.5 μg/mL (A549). IC_50_ was higher compared to that of ctrl 2. The EO was not toxic for HUVEC cells (IC_50_ = 196 ± 1.3 μg/mL) while ctrl 2 was highly toxic (IC_50_ = 0.2 μg/mL).	[123]
Egypt	fresh plants	EO	wi-38	Trans-menthone Pulegone Cis-menthone 1, 8-cineole menthol L-linalool, camphene, and sabinene	17.1% 16.2% 14.35% 9.11% 7.21%	The cytotoxic effect was very low.	[118]
-	flower	NPs with H_2_O ext.	A549 H358	-	-	↓viability. Cytotoxic effect at IC_50_ = 162.86 μg/mL (A549) and IC_50_ = 412.23 μg/mL (H358).	[124]

MeOH: methanol, H_2_O: water, ext.: extract, -: not specified, ctrl 1: paclitaxel at 10^−6^ μg/mL, CH_2_Cl_2_: dichloromethane, EO: essential oil, ctrl 2: doxorubicin 0.9 μg/mL, IC_50_: half-maximal inhibitory concentration, NPs: nanoparticles, and ↓: decreased.

**Table 5:** Effects of *Pinus sylvestris* L. on LC and normal cell lines.

Plant Origin	Plant Organ	Extract Type	Cell Lines	Bioactive Compounds	Bioactive Compound Concentrations	Effects	Reference
Estonia	needles	MeOH ext.	LU-1	-	-	Cytotoxic effect at IC_50_ = 54.62 ± 1.15 µL/mL	[49]
wood industry Pietarsaari	bark	H_2_O ext.	A549 IMR90	Sugars Phenolic compounds	-	Weak cytotoxic effect at IC_50_ > 2000 μg/mL	[135]
-	leaves	nanocomposites with H_2_O ext.	HEK293 A549	Polyphenols Carbohydrates Sugars Minerals	-	Cell growth inhibition at lower concentrations Cell death at higher concentrations Biocompatibility with HEK293 cells Cytotoxic effect against A549 cells	[136]

MeOH: methanol, ext. extract, -: not specified, IC_50_: half-maximal inhibitory concentration, and H_2_O: water.

**Table 6:** Effects of *Plantago major* L. on LC and normal cell lines.

Plant Origin	Plant Organ	Extract Type	Cell Lines	Bioactive Compounds	Bioactive Compound Concentrations	Effects	Reference
Taiwan	whole plant	H_2_O ext.	H520 H661	-	-	Cytotoxic effects at IC_50_ = 530 µg/mL (H661) and IC_50_ = 606 µg/mL (H520) These values were higher than those of ctrl 1	[140]
Indonesia	seeds, roots, leaves, and petioles	MeOH and H_2_O ext.	A549	Ursolic and oleanolic acids; aucubin	-	Seed ext.: better antiproliferative activity with IC_50_ = 153.38 ± 2.56 μg/mL (MeOH ext.) and IC_50_ = 290.79 ± 10.37 μg/mL (H_2_O ext.) MeOH ext.: IC_50_ = 343.97 ± 28.34 μg/mL (leaves), 392.14 ± 140.97 μg/mL (roots), and 475.49 ± 83.63 μg/mL (petioles) H_2_O ext.: IC_50_ > 1000 μg/mL (leaves, petioles, and roots) Ctrl 2: higher antiproliferative effect (IC_50_ = 0.09 ± 0.02 μg/mL)	[141]
Indonesia	seeds, roots, leaves, and petioles	-	A549	Ursolic and oleanolic acids; aucubin	-	Antiproliferative effect: ursolic acid (IC_50_ = 6.37 ± 0.53 μg/mL), oleanolic acid (IC_50_ = 29.37 ± 3.94 μg/mL), and aucubin (IC_50_ > 100 μg/mL) Ctrl 2: higher antiproliferative effect (IC_50_ = 0.09 ± 0.02 μg/mL)	[141]
Turkey	seeds and leaves	EtOH ext.	A-549 Beas-2B	-	-	Seed ext.: 78% toxicity against A549 (4 mg/mL); there was lack of toxicity against Beas-2B Leaf ext.: No cytotoxic effect	[142]
-	-	-	mice bearing 4T1 cells (mouse breast tumor cell line responsible for severe LM) CHO	PMS	-	PMS (300 μg/mL) did not affect the viability of CHO cells and had no side effects on normal cells Metastatic lungs foci: treated (29.5 ± 8.4) vs. control (92.8 ± 11.9) LM volume: treated (36% ± 7.5%) vs. control (69.3% ± 9%)	[143]
-	-	-	metastatic 95D cell line	PMS	-	↓ cell viability, proliferation, colony formation, stemness, invasion, migration, and mitochondrial membrane potential	[31]
-	-	-	A549	Linalool p-coumaric acid	-	Cytotoxic effect at IC_50_ = 438 ± 6.48 μM (linalool) and IC_50_ = 412 ± 5.50 μM (p-coumaric acid) vs. IC_50_ = 45 ± 2.50 μM (ctrl 1)	[144]

H_2_O: water, ext.: extract, -: not specified, IC_50_: half-maximal inhibitory concentration, ctrl 1: 5-fluorouracil, MeOH: methanol, ctrl 2: doxorubicin, EtOH: ethanol, CHO: Chinese Hamster Ovary, LM: lung metastasis, PMS: plantamajoside, and ↓: decreased.

**Table 7:** Protective effects of *Plantago major* L. extracts on renal toxicity induced by chemotherapy in rats.

Protective Extract, Posology	Incriminated Compound	Toxic Effect	Reference
Vitamin E (100 mg/kg) *Plantago major* extract (300, 600, and 1200 mg/kg) 20 days	Cisplatin	Nephrotoxicity Oxidative stress	[145]
Vitamin E (100 mg/kg) *Plantago major* extract (300, 600, and 1200 mg/kg) 5 days before and 2 weeks after cisplatin administration	Cisplatin	Renal dysfunction Tissue damage	[146]
Vitamin E (100 mg/kg) *Plantago major* extract (600 and 1200 mg/kg) 35 days	Doxorubicin	Nephropathy	[147]
*Plantago major* extract (600 and 1200 mg/kg) 5 weeks; doxorubicin was administered on the 7th day	Doxorubicin	Renal inflammation	[148]
*Plantago major* extract (600 and 1200 mg/kg) 5 weeks; 7 days before and 4 weeks after doxorubicin administration	Doxorubicin	Nephropathy	[149]
*Plantago major* extract (600 and 1200 mg/kg) 5 weeks; adriamycin was administered on the 7th day	Adriamycin	Renal fibrosis	[150]
*Plantago major* extract (600 and 1200 mg/kg) 5 weeks; adriamycin was administered on the 7th day	Adriamycin	Nephropathy	[151]

**Table 8:** Effects of *Sambucus nigra* L. on LC and normal cell lines.

Plant Origin	Plant Organ	Extract Type	Cell Lines	Bioactive Compounds	Bioactive Compound Concentrations	Effects	Reference
Egypt	flowers, leaves, and stems	EtOH extr.	A549	Phenolic compounds	-	Chlorogenic acid: cytotoxic at IC_50_ = 5.97 ± 0.27 μg/mL Stem ext.: cytotoxic at IC_50_ = 10.78 ± 0.61 µg/mL Leaf ext.: cytotoxic at IC_50_ = 75.09 ± 8.14 µg/mL	[159]
-	fruits	H_2_O ext.	A549 PBMC	Anthocyanins (C3S5G, C3,5diG, C3S, and C3G) and polyphenols (flavonols glycosides-rutin, and quercetin-3-O-glucoside).	-	No cytotoxic effect was observed on A549 cells ↑ proliferation of PBMC cells	[154]
Iran	shoots	H_2_O ext. incorporated in ZnO-CuO NPs	A549	-	-	Low toxicity; IC_50_ = 785 μg/mL At 1000 μg/mL, the cell viability was lower than that of the ctrl	[160]
-	-	NPRO Natural Products company plant ext. dissolved in DMSO	CCD-34Lu	-	-	IC_50_ not detected.	[73]
Portugal	berries	juice	NCI-H460 PLP2	Anthocyanins: C3S, C3G, C3S5G	560 µg/mL, 390 µg/mL, 140 µg/mL	Anticancer activity with IC_50_ = 16 ± 1 µg/mL (NCI-H460) Very weak toxicity against PLP2 cells (IC_50_ > 400 µg/mL)	[161]

EtOH: ethanol, ext: extract, IC_50_: half-maximal inhibitory concentration, -: not specified, C3S5G: cyanidin-3-O-sambubioside-5-O-glucoside, C3,5diG: cyanidin-3,5-di-O-glucoside, C3S: cyanidin-3-O-sambubioside, C3G: cyanidin-3-O-glucoside, ↑: increased, PBMC: peripheral blood mononuclear cells, NPs: nanoparticles, ctrl: doxorubicin at 4 and 8 μg/mL, DMSO: dimethyl sulfoxide, and PLP2: normal liver primary cells.

**Table 9:** Effects of *Thymus serpyllum* L. on LC and normal cell lines.

Plant Origin	Plant Organ	Extract Type	Cell Lines	Bioactive Compounds	Bioactive Compound Concentrations	Effects	Reference
produced by the company “Natures” (batch number: 2813/2012)	-	EO	NCI-H460	Thymol	38.5%	Cytotoxic effect at IC_50_ = 37.17 ± 3.18 μg/mL.	[165]
Montenegro	herb	EO	A549 MRC5	p-cymene, geraniol, linalool, geranyl acetate, 4-terpineol, and borneol	19.04%, 11.09%, 9.16%, 6.49%, 5.53%, and 5.24%	Cytotoxic effect at IC_50_ = 0.36 ± 0.02 µL/mL (A549), IC_50_ = 0.33 ± 0.03 μL/mL (MRC5)	[25]
-	-	-	A549	Thymol Geraniol Nerol acetate	-	Inhibition of migration: thymol > nerol acetate > geraniol	[168]

-: not specified, EO: essential oil, IC_50_: half-maximal inhibitory concentration, and >: more than.

**Table 10:** Effects of *Tussilago farfara* L. on LC cell lines.

Plant Origin	Plant Organ	Extract Type	Cell Lines	Bioactive Compounds	Bioactive Compound Concentrations	Effects	Reference
Purchased from Shanxi, China	flower buds	PE ext.	LLC cells	Tussilagone, 7β-(3′-Ethyl-cis-crotonoyloxy)-1α-(2′-methylbutyryloxy)-3(14)-dehydro-Z/Enotonipetranone	-	Antiproliferative effects at IC_50_ = 105.13 ± 1.34 μg/mL The minimal inhibition rate: 3.86% The maximum inhibition rate: 96.79%	[173]
China	flower buds	EtOH ext.	A549	C_24_H_34_O_6_ (1 and 2), C_20_H_28_O_4_ (3), C_20_H_26_O_4_ (4), C_22_H_31_ClO_7_ (5), C_20_H_30_O_4_ (6), C_23_H_32_O_6_ (7), C_23_H_34_O_6_ (8), C_12_H_16_O_3_ (9 and 10), C_13_H_14_O_5_ (11), tussfararin F (12), altaicalarin B (13), 7β-(3- ethyl-cis-crotonoyloxy)-1α-(2-methylbutyryloxy)-3,14-dehydro-Z-notonipetranone (14), teucladiol (15), 4(15)-eudesmene-1β,6αdiol (16), and eudesma-4,11-diene-1β,15-diol (17)	-	Antiproliferative effects at IC_50_ = 19.4 ± 0.87 µM (compound 6), IC_50_ = 12.6 ± 0.81 µM (compound 8), IC_50_ = 9.04 ± 0.58 µM (compound 14), and IC_50_ = 0.73 ± 0.06 µM (ctrl 1) 14.27% total apoptotic cells (compound 14 at the lowest concentration) vs. 7.80% (ctrl 1) Compound 14: G2 and S phases of cell cycle changes	[174]
Purchased from Shanxi, China	flower buds	complex procedure to obtain TFPB1	A549	TFPB1	-	Antiproliferative effect; 54.4% inhibition (1000 μg/mL—tested concentration of isolated compound)	[19]

PE: petroleum ether, ext.: extract, IC_50_: half-maximal inhibitory concentration, EtOH: ethanol, ctrl 1: doxorubicin, and TFPB1: polysaccharide from flower buds of *Tussilago farfara* L.

## Data Availability

No new data were created or analyzed in this study. Data sharing is not applicable to this article.
